# Tensor decomposition of TMS-induced EEG oscillations reveals data-driven profiles of antiepileptic drug effects

**DOI:** 10.1038/s41598-019-53565-9

**Published:** 2019-11-19

**Authors:** C. Tangwiriyasakul, I. Premoli, L. Spyrou, R. F. Chin, J. Escudero, M. P. Richardson

**Affiliations:** 1 Department of Basic and Clinical Neuroscience, Institute of Psychiatry, Psychology and Neuroscience (IoPPN), King’s College London, London, UK; 2 School of Engineering, Institute for Digital Communications, The University of Edinburgh, Thomas Bayes Rd, Edinburgh, EH9 3FG UK; 3 Muir Maxwell Epilepsy Centre, Centre for Clinical Brain Sciences and MRC Centre for Reproductive Health, The University of Edinburgh, 20 Sylvan Place, Edinburgh, EH9 1UW UK

**Keywords:** Epilepsy, Biomedical engineering

## Abstract

Transcranial magnetic stimulation combined with electroencephalography is a powerful tool to probe human cortical excitability. The EEG response to TMS stimulation is altered by drugs active in the brain, with characteristic “fingerprints” obtained for drugs of known mechanisms of action. However, the extraction of specific features related to drug effects is not always straightforward as the complex TMS-EEG induced response profile is multi-dimensional. Analytical approaches can rely on *a-priori* assumptions within each dimension or on the implementation of cluster-based permutations which do not require preselection of specific limits but may be problematic when several experimental conditions are tested. We here propose an alternative data-driven approach based on PARAFAC tensor decomposition, which provides a parsimonious description of the main profiles underlying the multidimensional data. We validated reliability of PARAFAC on TMS-induced oscillations before extracting the features of two common anti-epileptic drugs (levetiracetam and lamotrigine) in an integrated manner. PARAFAC revealed an effect of both drugs, significantly suppressing oscillations in the alpha range in the occipital region. Further, this effect was stronger under the intake of levetiracetam. This study demonstrates, for the first time, that PARAFAC can easily disentangle the effects of subject, drug condition, frequency, time and space in TMS-induced oscillations.

## Introduction

Transcranial magnetic stimulation (TMS) is a non-invasive tool to probe neurophysiological processes in the human brain. A TMS pulse depolarizes the stimulated neuronal population and remote anatomically connected regions^1^. The registration of TMS effects with electroencephalography (EEG) allows to quantify and characterize spread of neural activation that follows in time, spatial and frequency domains^2^. The summation of synaptic potentials produces a series of time-locked positive and negative deflections visible in the EEG signal, termed the TMS-evoked potentials (TEPs). TEPs are a sequence of peaks which reflect cortical reactivity and changes in their amplitude and latency reflect changes in cortical activity^3^. In addition, brain responses to TMS can be interrogated applying a time-frequency analysis at single trial level removing the evoked (i.e. TEPs) component from the signal. TMS-induced oscillations are the result of this analytical approach and they provide non-phase locked neural information^4^.

TEPs and TMS-induced oscillations are outcome measures used to characterise brain states in health, diseases and under experimental conditions such as drug manipulation^5^. Previous work showed that TMS-EEG is a powerful tool to investigate effects of drugs acting in the human brain^6–10^. In these studies, the effects of drugs were quantified in term of differences between conditions (or subjects) in evoked activity in specific time windows corresponding to TEPs and in specific sets of EEG electrodes. A cluster based permutation approach is the golden standard used to overcome the problem of multiple comparisons. It requires an a-priori selection of time windows or a post-hoc correction for the large number of non-independent comparisons across many tested conditions. It seems highly likely that important effects will be lost through inadvertent selection of the “wrong” time windows and/or electrodes, or through the necessarily harsh post-hoc correction for multiple non-independent comparisons.

The high dimensionality of TMS-EEG data is a challenge for analysis and interpretation, and motivates approaches to simplify the data by reducing the dimensionality. Specifically, we can hypothesise that TMS stimulation of the brain gives rise to activity in specific brain networks following stimulation, and that these networks will have a specific spatial distribution and specific spectral characteristics (i.e. the network operates in a particular frequency range) – but identifing such underlying patterns in highly multidimensional data is difficult. Here, we apply a methodology based on tensor decomposition to reveal such underlying patterns.

The term “tensor” refers to a multi-way (i.e. multidimensional) array, that is a collection of variables that can be indexed by more than two terms. Whereas the position of and element in a vector or matrix is determined, respectively, by one (e.g., *i*) or two indices (e.g., *i*, *j*), the values in a tensor are indexed by more than two parameters: *i*, *j*, *k*...^11^. In a similar way to how matrix decompositions (e.g., principal component analysis) can represent a two-dimensional array (a matrix) as a product of factor matrices, tensor decompositions allow us to extract from seemingly complex multidimensional data parsimonious and unique representations of underlying patterns^11,12^. Since the introduction of the PARAFAC algorithm, which decomposes a tensor into a sum of outer products of low-rank components^13^, tensor decompostions, and PARAFAC in particular, have been used in a wide range of studies of EEG activity^14^. Seminal studies focused on the analysis of event-related potentials^15,16^. Subsequent tensor decompositions of EEG data enabled the inspection of time-frequency representations of EEGs during cognitive states^17^. Tensor decomposition has also been used in artefact rejection and estimation of seizure onset zone^18,19^. Other applications include localisation of EEG sources^20^, connectivity estimation^21^, brain computer interfaces^22,23^, and feature extraction in clinical and psychological studies^24–26^. Tensor decomposition are also useful when fusing EEG with other datasets^27–29^. Overall, the use of tensor decompositions is advatageous over matrix factorisations when the data are naturally multdimensional like in the case of EEG, and TMS-EEG^12^.

In this study, we sought to apply a data-driven approach, exploting the multidimensional structure of previously collected TMS-EEG data, allowing a parsimonious dimensionality reduction that summarises effects in the high-dimensional data. We hypothesise that PARAFAC will be able to reveal underlying patterns of activity with different topographical (accounting for the spatial distribution of a brain network), temporal (indicating time period after TMS stimulation during which the network is active) and spectral (informing about the typical operating frequency of the network) profiles that will be characteristic of the effects of each type of anti-epileptic drug (AED) in the TMS-EEG data without a-priori assumptions.

## Methods

### Subjects

Thirteen healthy male volunteers aged 19–34 years (mean age 25.2 years, SD = ±4.62) participated in the study after written informed consent was given. All subjects were classified as right-handed according to the Edinburgh Handedness Inventory^30^ and underwent physical examination and screening for any contraindications to TMS or study drugs^31^. The College Research Ethics Committee (CREC) of King’s College London approved the research, which was performed in accordance with relevant guildlines and regulations. Informed consent was obtained from all participants. The TMS-evoked EEG potential (TEP) analyses of this sample have been published previously^7,32^.

### Experimental design

We performed a double-blind, randomized, placebo-controlled, crossover study to investigate the impact of levetiracetam (LEV, 3000 mg) and lamotrigine (LTG, 300 mg) on TMS-induced EEG oscillations. Each subject participated in three experimental sessions in total, administered lamotrigine, levetiracetam or placebo in each session in a randomized order, spaced at least one week apart to allow a washout period. At each session, we first performed baseline pre-drug TMS-EEG recording. Later, the post-drug recording was performed two hours after drug ingestion, please see the details of the experimental setting and protocol in the Supplementary Section A.1.

### Data analysis

#### TMS-EEG data construction

TMS-induced oscillations were analysed using MATLAB® (Mathworks Ltd, USA, R2012b) (The Mathworks Inc.) and FieldTrip toolbox^33^. After excluding records/trials with prominent eye movements, blinks, and muscle artefacts (on the basis of visual inspection), EEG data was analyzed using an established multistep procedure^34^. Data was down sampled to 1 kHz, segmented 1 s before and after the pulse, and linearly interpolated for ±10 ms to remove the TMS artefact. Bad channels were removed from the EEG, and the signal was reconstructed by interpolating the surrounding electrode signals. Data was then notched filtered (50 Hz). Independent Component analysis (ICA) was applied to remove TMS-related artifacts (i.e., the cranial muscle response, the recharging of capacitors, and related exponential decay artifacts^35–37^, as well as further muscle and ocular activity. Finally, remaining data were re-referenced to the average of all electrodes, baseline corrected (from −1000 to −50 ms) and band-pass filtered (1–80 Hz).

After that, for each segment we estimated its time-frequency plot by applying a Hanning taper windowed fast Fourier transform (FFT) with frequency-dependent window length (width: 3.5 cycles per time window, time steps: 10 ms, frequency steps: 1 Hz from 4 to 45 Hz)^38^. TMS-induced responses were obtained by subtracting the individual time-domain average from each trial before calculating the TF of the single trials^39^. We performed single-trial normalization by z-transforming the TF of each trial for each frequency. The z-transformation was based on the respective mean and standard deviation derived from the full trial length. This was followed by an absolute baseline correction for each trial, by subtracting the average of the 100 to 50 ms period for each frequency to ensure z-values represented a change from pre-TMS baseline.

At the end, we had an array of 61 × 42 × 201 elements (61 channels, 4 Hz to 45 Hz with frequency resolution of 1 Hz, and data starting from −1000 to + 1000 ms with time step of 10 ms). Note that, to minimise TMS and DC shifts effects along the time (3^rd^ dimension) and frequency (2^nd^ dimension) axes, we selected the data starting from 40 ms after the TMS pulse to 1000 ms after the TMS pulse and frequency bins between 4 to 34 Hz resulting in a new 3D array of 61 × 31 × 98 elements. These steps were repeated for all segments and all channels.

#### Tensorisation of TMS-EEG data and PARAFAC modelling

**Figure 1:**
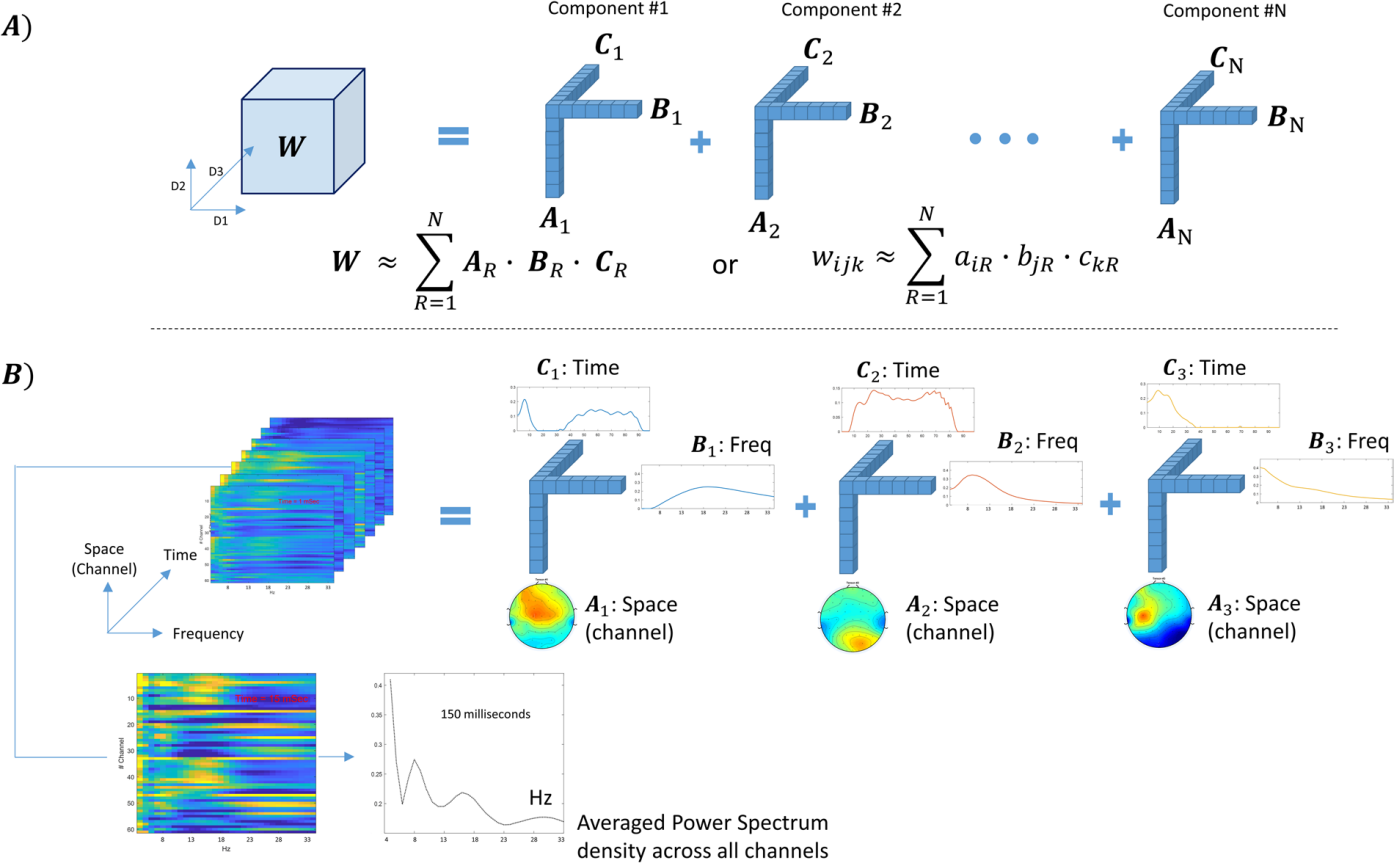
(**A**) Shows N decomposed components from the 3D tensor **W**, each component comprises three vectors (A, B and C). (**B**) shows an example when the technique was used to decompose the 3D (space × frequency × time) of subject#2 post LEV. In this case, the three components represent high, medium and low-frequency ranges (15–30 Hz “Beta”, 6–13 Hz “Alpha”, and 4–6 Hz “Theta”. The bottom two insets show a Space-vs-Frequency plot at 150 milliseconds after TMS pulse and its grand average across all channels.

The TMS-EEG data construction described in section 2.3.1 resulted in a three dimensional tensor [channel (or space) × frequency × time], representing a time varying spectrum of all channels. Tensors are multi-dimensional data arrays that extend vectors (one dimensional) and matrices (two dimensional) to more than two dimensions^11,12^. This three-dimensional tensor [channel (or space) × frequency × time] will be used in our subsequent tensor decomposition based analysis. Figure 1 illustrates the principle of tensor decomposition based on the PARAFAC model for our tensorised TMS-EEG data (as a 3D tensor for simplicity).

Assuming that we have a 3D tensor **W**, this data array can be approximated as a sum of *N* rank-one tensors, which represent underlying components^13,17^. Each component is an outer product of three matrices (**A**, **B** and **C**) as:

1 $${w}_{ijk}\approx \mathop{\sum }\limits_{r=1}^{n}{a}_{ir}\cdot {b}_{jr}\cdot {c}_{kr}$$

where *w*_*ijk*_ is an element in the tensor **W**, which is approximated by the summation of *N* rank-1 components which are the outer product of ${a}_{r},{b}_{r},{c}_{r}\,$, where, for example, ${a}_{ir}$is an element in the matrix **A** which contains the profiles of the extracted components along the first dimension (channel or space) in its columns **a**_r_. Likewise, **B** and **C** contains the estimated components along the second (frequency) and third (time), respectively, see Fig. 1(A,B). This data model assummes that the neural generators resulting in the scalp EEG activity are stationary during the recording period.

#### Selecting the optimum number of components

There is no a priori means to determine how many components will best represent the data. Explained varinace were used to help estimate an appropriate number of components in PARAFAC.

To estimate the optimal number of components, we decomposed a 5D tensor (consisting of all conditions from all subjects [61 × 31 × 98 × 13 × 6 elements]) into a different number of components ranging from one to eight (*n* = 1, .., 8 in Eq. 1), and estimated the explained variance in each instance. The selection of a relatively low number of components reduces the chances of overfitting and faciltiates its intepretation. More importantly, the topographical, temporal and spectral profiles of the extracted components were inspected to determine a number of PARAFAC components that would aid in the interpretation of the data. It is important to inspect the profiles of components extracted for each considered value of *n* since it is not guaranteed that the components extracted when computing PARAFAC with *n*–1 will appear again when doing so with *n* components^13^.

Besides estimating the explained varaince, we also estimated the core consistency diagnosis (CORCONDIA, see the Supplementary Section A2)^40^. CORCONDIA is a heuristic measure to check if the data can be modelled fully multilinearly.

#### Using tensor decomposition to characterise and contrast effects of AEDs and placebo

**Figure 2:**
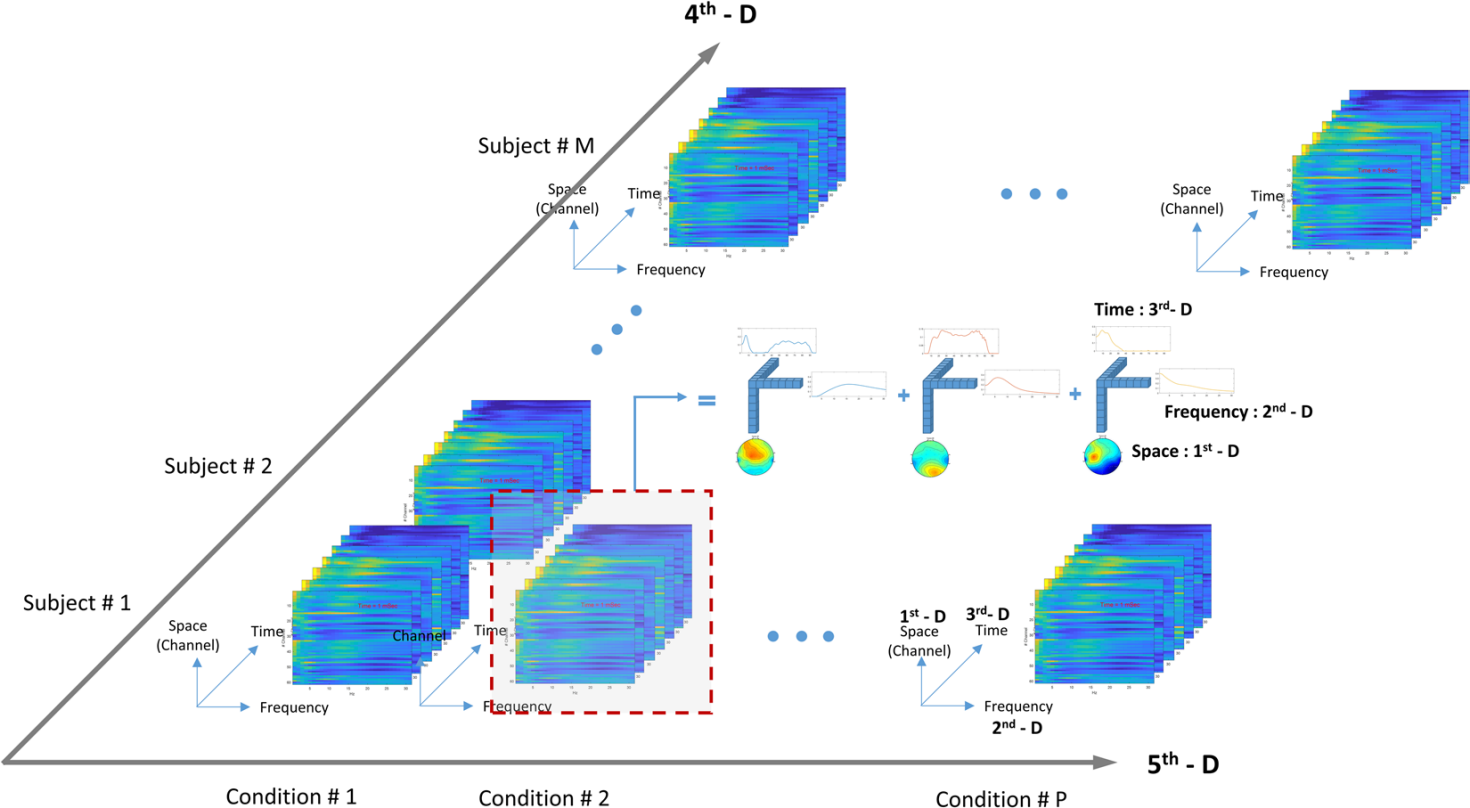
The five-dimensional tensor in this study comprises of space (channel), frequency, time, subject and condition (1^st^, 2^nd^, 3^rd^, 4^th^ and 5^th^ dimension, respectively).

Building on the 3D tensor described above, we constructed a five dimensional tensor consisting of the three previously described dimensions (space, frequency, time) and adding two further dimensions, subject and condition (see Fig. 2), by stacking 3D tensors obtained from section 2.3.2 in order to account for all the interactions of space, time and EEG oscillation frequency with the effects of drugs on the subjects. We then tested the effects of drugs on the subjects by contrasting conditions in four different ways, and including these conditions in the 5^th^ dimension (condition):

Model 1: We also use this model as a proof of concept to study the components obtained from PARAFAC without any effect from drug in order to validate the tensor decomposition of TMS-EEG data.

Model 2: To test the hypothesis that levetiracetam and lamotrigine have different effects, we included four conditions: pre-LEV, post-LEV, pre-LTG, post-LTG.

Model 3: To test the hypothesis that levetiracetam has a different effect than placebo, we included four conditions: pre-placebo, post-placebo, pre-LEV, post-LEV.

Model 4: To test the hypothesis that lamotrigine has a different effect than placebo, we included four conditions: pre-placebo, post-placebo, pre-LTG, post-LTG.

These four separate models allow us to first validate the application of PARAFAC to TMS-EEG data and then to compare in pairs the effect of each drug between them and versus placebo in data-driven, unsupervised way. Considering the post processed data (3D tensors) obtained from step 2.3.2, for each subject (per condition) we had a data array of 61 × 31 × 98 elements. After stacking these 3D tensors from all subjects for the specific conditions as described in each model, we obtained a 5D tensor of 61 × 31 × 98 × 13 × 4 elements. Unlike the example in Fig. 1, showing the decomposition of the 3D tensor, with the newly constructed 5D tensor we could decompose this 5D array into a sum of 5 rank-one tensors (space (or channel), frequency, time, subject, and condition).

In this study, we used the N-way toolbox version 3.3 for tensor decomposition^41^ (http://www.models.life.ku.dk/nwaytoolbox). Note that we applied the non-negativity constraint to all dimensions while performing decomposition. Thus every element in the decomposed arrays would be at or greater than zero^14,17,42^. This constraint was imposed for a ease of interpretation.

#### Statistics

We applied a permutation based analysis to test for significant difference between pre-vs-post drug. All steps taken are presented as follows (see the graphical representation of all the steps in the Supplementary Fig. A1). At each model, we first decomposed the 5D tensor (with 61 × 31 × 98 × 13 × 4) into three components. These components were considered as ‘master’ components (that is, the ‘true labelled’ components, in distinction to permuted components, see below). Each of these components consisted of five profiles across the five dimensions (axes). For example in model-1, at each component we obtained five rank-1 tensors with 61, 31, 98, 13, and 4 elements for space, frequency, time, subject and condition, respectively.

Then we permuted this 5D tensor for 1,000 iterations. At each iteration, we permuted the elements on the 4^th^ and 5^th^ dimensions (subjects and conditions). Next, we decomposed this permuted 5D tensor while fixing all elements in the first three tensors. From this step, we obtained a new set of five rank-1 tensors, where thre first three tensors (representing space, frequency and time) were similar to the ones in the master, but the elements in the 4^th^ and 5^th^ tensors could be different from the master because shuffling the data along those dimensions destroys the inherent structure.

To assess the effects after drug/placebo intake, we subtracted the value on the 5^th^ dimension of the pre drug from the value post drug. For example, considering the 2^nd^ component of the master (true label), we subtracted the value before LEV intake from the value after LEV intake see the Supplementary Fig. A1). For the permuted data, at each iteration we estimated the level of change post drug as we did with the master. Then, we computed a histogram of these values. The level of change in master (true label) was significant if its value was less (or greater) than 2.5% of the distribution of the histogram. Note that the green square in the histogram at the bottom tight of the Supplementary Fig. A1 represents the difference between pre-vs-post LEV, where the two red vertical lines represent the upper and lower 2.5% of the histogram.

## Results

### Optimum number of decomposed components

We first explored our data to determine the optimal number of components. We decomposed the 5D tensor (space, frequency, time, subject, condition) build from all subjects and all six conditions into a range of number of components from one to eight. First, we showed the percentage of explained variance at different number of components in Table 1. Then, we showed the topographical, temporal and spectral profiles of the extracted components from all eight cases (see the Supplementary Figs. A2–A4).

**Table 1:** Percentage of explained variance by a number of decomposed components.

Scenario	No. of	Explained
	Components	variance (%)
I	1	29.14
II	2	34.06
III	3	39.33
IV	4	41.21
V	5	42.53
VI	6	43.49
VII	7	44.05
VIII	8	44.03

From Table 1, a marked change was found in these parameters when increasing the number of components from one to three: i.e. ~10% increase in terms of explained variance. Above 4 components, further increasing the number of components did not significantly change either of these parameters.

In the Supplementary Fig. A2, representing the decomposed components on the 1^st^ dimension (space), we found three typical underlying spatial patterns (highlighted in green, red and blue), which were relatively consistent (at least 6 out of 8 scenarios).

Moving on to the frequency axis (2nd dimension), if we decomposed the 5D tensor into a single component, this component would be represented primarily in the alpha range, see the Supplementary Fig. A3. When decomposing the same 5D tensor into two components, a component primrily in the theta frequency range was found in addition to the alpha component. Decomposed into three components, we observed they were distinct, primarily in theta, alpha and beta bands. After that, increasing the number of components did not add any other distinct components at other frequency bands, as most of the further decomposed components overlapped with the components found when decomposed into just three.

Finally, in the time axis unlike the first two axes it was harder to justify a number of independent components. By visual inspection, at least three distinct components were found, see the Supplementary Fig. A4.

Taking these together, we decided to decompose the 5D tensor into three components where the three distinct frequency band and three unique spatial patterns were clearly observed and the explained variance reached its plateau at about 40%.

### Comparison across the four models

**Figure 3:**
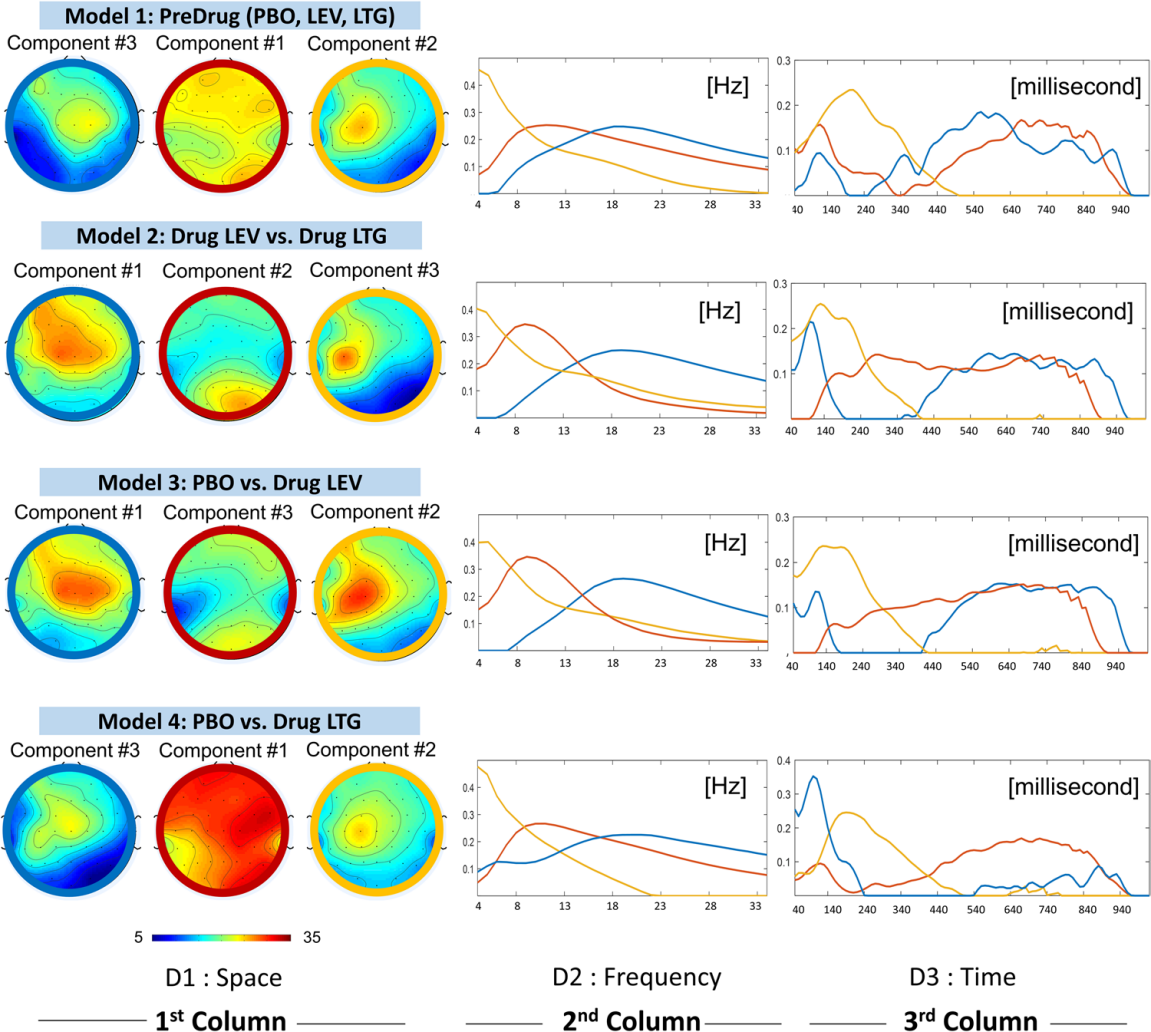
We present the three components decomposed from 5D tensor in four different models. The top row shows the decomposed components in space (topographical plots), frequency and time dimension. Three colours (blue, red and yellow) are used to indicate the three decomposed components: beta (with peak frequency between 15–30 Hz), alpha (with peak frequency between 6–13 Hz) and theta (with peak frequency between 4–6 Hz), respectively.

Figure 3 shows three components decomposed from our four different models. The 5D tensor from each model was decomposed into three components at three different frequency bands (theta, alpha, and beta, see the 2^nd^ column in Fig. 3).

The model 1 is a proof of concept showing the three physiological components (beta, alpha and theta) decomposed from the TMS-EEG data without any effect from LEV or LTG.

First considering the beta components (labelled in blue) from all models, these components mostly represented frontal brain activities. On the time axis (3^rd^ dimension), each of these beta components could be divided into 3 phases: (1) initial peak (during 40–200 milliseconds), (2) suppression (200–400 milliseconds) and (3) rebound (400 milliseconds onward). When we compared between models 3 and 4 (between LTG and LEV), one could observe less rebound of this beta component in LTG as compared to LEV.

The next component, which was predominantly observed in alpha range, showed the most variability (in terms of magnitude and spatial pattern) among the three components (theta, alpha, and beta) across all models. Whereas one could see the alpha component dominating occipital lobe in models 2 and 3, in model 4 this alpha activity can be seen everywhere (with high amplitude) except on the areas next to the earlobes.

Moving on to the last component, or theta labelled in orange, it was spatially identical across all models and represented the activities on C3. This component reached its peak around 200 milliseconds and completely suppressed starting ~400 milliseconds to the end of each recording.

### Model 2: comparison of the effects of levetiracetam and lamotrigine

**Figure 4:**
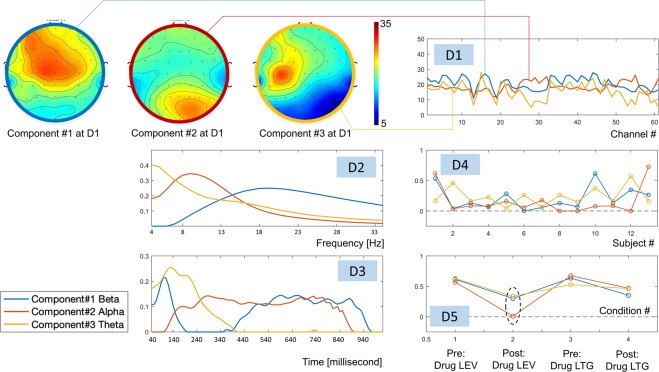
The three components decomposed from the 5D tensor in all subjects during pre-drug and post-drug conditions with LEV or LTG. Note that: D stands for Dimension.

Figure 4 shows the three decomposed components in five dimensions, which were highlighted in blue, red and orange for 1^st^, 2^nd^ and 3^rd^ components respectively. The first component (blue) represented the brain activities (in the beta range, peak at 19 Hz) over the frontal and central areas. On the time axis (3^rd^ dimension), this component initially peaked at ~90 milliseconds after applied TMS pulse, then suppressed between 190–400 milliseconds, and rebounded from 440 milliseconds to the end of the recording.

The second component (red) represented the activities with relatively lower frequency (at alpha band or between 6–13 Hz), which predominantly involved the occipital lobe. Initially, during 40–140 milliseconds after the TMS pulse, while the 1^st^ component (frontal beta) was reaching its peak, this component (occipital alpha) was absent. Subsequently, during 140–340 seconds, while the 1^st^ component was declining and eventually completely diminished, this 2^nd^ component was on the rise and reached a plateau. Starting from 440 milliseconds until the end of the recording, these two components coexisted.

The last component (3^rd^, orange) was found in the theta band (4–6 Hz), centered on EEG electrode C3, which was the location where the TMS pulses were given. This component reached its peak between 90–240 milliseconds, and later was suppressed starting from 440 milliseconds until the end of recording (which was the period where both 1^st^ and 2^nd^ components coexisted).

**Figure 5:**
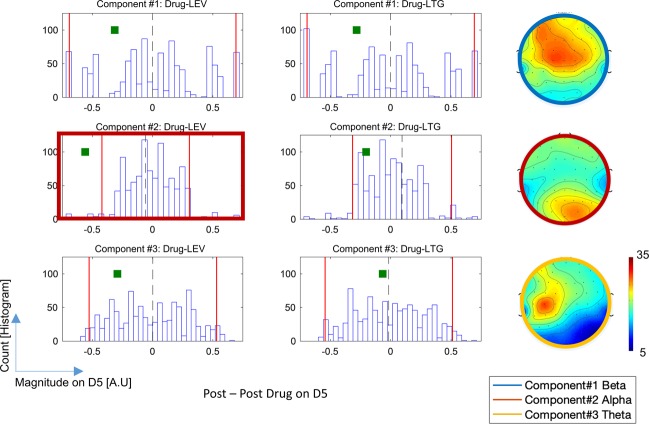
Each histogram shows the distribution of strength on the 5^th^ dimension obtained from 1000 iterations of permutation. Two vertical red lines indicate the upper and lower 2.5% of the histogram. Each green square oindicates the difference between pre and post medication on the 5^th^ dimension. In the top and bottom rows (showing the results from components 1 and 3, respectively), no significant reduction was found. For component #2 (middle row), we observed the significant reduction in terms of strength on the 5^th^ dimension after receiving LEV.

Inter-subject variabilities were revealed in the 4^th^ dimension showing the 1^st^, 10^th^ and last subject being different from the others. On the 5th dimension condition (drug) effects were revealed, and we observed reduction in all components after receiving medication (both LEV and LTG). From this plot, one could see a stronger post medication effect for LEV as compared to LTG, especially in the 2^nd^ component. At a group level, there was a significant effect of reduction of the 2^nd^ component after LEV intake (see Fig. 5).

### Statistics

**Figure 6:**
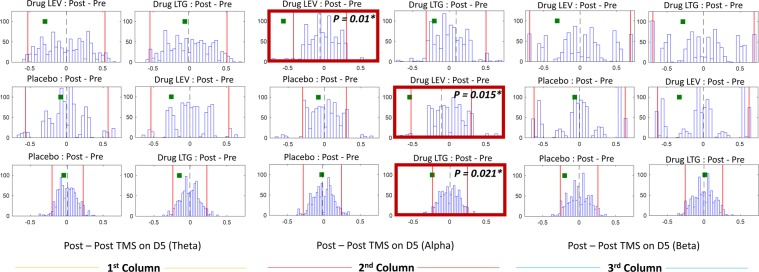
Each histogram shows the distribution of difference between pre and post medication(or placebo) from 1000 iteration. The two red lines in the histograms indicate the first and last 2.5 percent. The green square represents the post vs pre difference on the 5^th^ dimension. ^*^Denotes significant (P < 0.025). All histograms on the top, middle and bottom rows are the distribution from model 2, 3, and 4, respectively. Note that all the p-values in this study are reported in the Supplementary Table A1.

Figure 6 shows distributions of difference between pre-and-post medication from 1000 iterations in models 2, 3 and 4. Statistically, no significant change in either theta or beta components was found (see columns 1 and 3). Considering models 3 and 4, when we investigated the effects after drug vs after plecebo, we found significant reduction of alpha component in both post LEV (p = 0.015*) and post LTG (p = 0.021*) conditions. In model 2, when we compared the effects after drug intakes in both LEV vs LTG, the post-LEV shows a significantly stronger reduction of the alpha component than post-LTG with p = 0.01*.

## Discussion

In this study, we introduced a tensor decomposition method to reduce multi-dimensionality of TMS-EEG data. We showed a series of components which provides a parsimonious description of neurophysiological responses underlying TMS-induced oscillations. In addition we demonstrated the utility of PARAFAC on existing data to disentangle the effect of anti-epileptic drugs on TMS-indueced oscillations. This method does not require *a-priori* selection of anatomical regions of interest, time periods of interest and frequency components of interest in the multi-dimensional EEG data, and without requiring potentially harsh post-hoc statistical correction for multiple comparisons. PARAFAC revealed an effect of both levetiracetam and lamotrigine, significantly suppressing oscillations in the alpha range in the occipital region, during the time period approximately 140 ms − 840 ms after the TMS pulse. Furthermore, this technique also reveals that the suppression of alpha oscillations is significantly stronger during the intake of levetiracetam than lamotrigine.

### Optimum number of decomposed components and justification of PARAFAC model

From Table 1, the explained variance in the data reached its plateau when splitting into three components. This suggsests three as the optimal components. To justify our choice, we also visually inspected the decomposed profiles on space, time and frequency axes (Supplementary Figs. A2–A4). This is important as the profiles of components extracted for each considered value of n since it is not guaranteed that the components extracted when computing PARAFAC with *n*–1 will appear again when doing so with *n* components^13^. The fact that similar patterns appeared naturally provides further support to the interpretability of our chosen model. The inspection of the components also enabled us to grasp which physiological processes captured by the data-driven components were prominent in the TMS-EEG recordings. The results support our choice of 3 components. It was clear that along the frequency axis (Supplementary Fig. A3) regardless of the number of decomposed components we could only break down into maximum three different frequency bands (theta, alpha, and beta). Moving on to the spatial axis (the Supplementary Fig. A2), it was debatable if a maximum number of components could be either three or four. Finally, along the time axis (the Supplementary Fig. A4), the optimal number of component was unclear (it could either be any number between two to four). Looking at the CORCONDIA in the Supplementary Table A2, we found that by extracting more than one component the value of CORCONDIA drop to zero. This suggests our 5D tensor is not a fully multilinear form and also explains the non-equivalent number of optimal components along different axes^40^. Taking all these together, we then decided to decompose into three components, where all three unique signatures along frequency and spatially domains were found, and the explained variances reached its plateau. It is important to note that the explained variance may not increase significantly with the number of components since the multilinear PARAFAC model will not explain the noise and random variations in the data. Furthermore, increasing the number of components would lead to higher computational cost^43^.

Although another family of tensor decompositions (Tucker decompositon) may provide a solution to the case with a non-equivalent number of optimal components, it does not preserve one-to-one interaction^33,44^. Hence, the decomposed components using Tucker decomposition are harder to interpret. To sum up, we decided to decompose the TMS-EEG 5D tensor using PARAFAC, which preserves one-to-one interaction. That is each component will be entitled to a unique interpretation, for example, the TMS induced component may be seen in a particular frequncy range, anatomical distribution and time period. Future work will explore the suitability of other more flexible, but still unique models such as PARAFAC2^21^, to improve the modelling of TMS-EEG data and reveal even more subtle interactions.

### Physiological meanings behind the three components

From model 1 (without drug) we found three physiological components (theta, alpha and beta), these components are highly similar (spatially, temporally and spectrally) across four models, see Fig. 3. Given that the PARAFAC solution is unique under very mild conditions, this further reinforces that the extracted components have physiological meaning. These components (frontal-sensorimotor beta, posterior alpha and theta related to the site of stimulation) represented the hidden signature of the data for all conditions (pre/post PLA, pre/post LEV, pre/post LTG). We considered the frontal-sensorimotor beta component to represent the spreading of cortical reactivity from the stimulated site (C3) through its neighboring areas via local fibers as well as to the contralateral motor cortex via corpus callosum^45^. Sensorimotor rhythms, which dominate the motor cortex, are found in mu (8–13 Hz) and beta rhythms (15–30 Hz)^46,47^. By giving a TMS pulse, it may elicit similar effects on the cortical neurons seen as event-related (de)-synchronization (ERD/ERS) time locked to motor movement over motor cortex areas^48–50^. Since the rebound of sensorimotor rhythms (synchronization) is uniquely observed in the beta range after giving stimuli^48,49^, by imposing the non-negativity constraint to the 5D tensor we might limit the decomposed component at this area only in the beta band. Moving on to the posterior alpha, it is found to be a key component shown to differentiate between the two drugs, seen as the stronger reduction of alpha component after LEV compard to LTG intake, in model 2. Considering both models 3 and 4 (placebo vs each type of drug), we found the significant reduction of this component after both LEV and LTG intake (whereas no change was found post placebo). Results suggest that both types of drug cause similar effects on the generation of posterior alpha. The same observation derived from the investigation of these AEDs on TEPs, where despite the varying profile of effects and regardless of the (putative) molecular targets of the different drugs, systemically administered LEV and LTG exert similar modulation of TEPs^9^. In addition, the effect on alpha was stronger under LEV exposure which had the highest average concentration in blood outside the reference range, with LTG averaging toward a lower concentration for its reference range^9^.

Lastly, considering theta, this component represents the TMS-induced effect on the stimulation site, because its spatial pattern was centred on C3 and its temporal signature shows a peak soon after stimualtion and then subsided.

### Strengths and weaknesses of this study

Unlike a conventional TMS-EEG analysis, which requires predefining time, anatomical area and frequency of interest, tensor decompositions offer a purely data-driven approach. In particular, we applied PARAFAC due to its parsimony and ease of interpretation since the interactions of the components are restricted. In our analysis, the 5D tensor for each mode had dimensions 61 × 31 × 98 × 13 × 4 (9,636,536 entries in total). Decomposing it with PARAFAC, the method was able to account for approximately 40% of the explained variance with just 3 components which include only 621 elements – i.e., 3 × (61 + 31 + 98 + 13 + 4), less than 0.01% of the total number of entries in the tensor. The results were tested under permutation-based statistics. We successfully showed that each decomposed component represents the unique signature on the spatial, spectral and temporal domains with physiological meaning. Furthermore, along the 4^th^ dimension, one could make the inference about these hidden signatures at a single subject level. Despite the postive results provided by this innovative analysis approach of TMS-EEG data, it must be take into consideration that the TMS pulse can induce unwanted somatosensory input that have an impact on TEPs^51^. We purposely selected a PARAFAC model with non-negativity constraints to simplify the interpretation of the components extracted from TMS-EEG activity in this first application of tensor decompositions to this type of data. However, we acknoledge that the choice of the non-negativity constraint implies that we were not able to reveal potential patterns in negative values and that our results are also limited by the small number of participants and we advice the reader to intepret them with care.

## Conclusion

To our knowledge, it is the first time tensor decomposition has been applied in TMS-EEG data. Our results show the power of tensor decompositions to reveal the profiles underlying the complex responses in TMS-EEG data associated with different AEDs in healthy subjects in a data-driven and parsimonious way. Future work will seek to develop classifiers able to predict the level of response to each AED in new subjects by projecting their TMS-EEG recordings on the “characteristic filters” associated with previously revealed tensor components in space, time and frequency^26,52^. We will also consider the possibility of applying tensor decompositions to TMS-EEG signals in the time domain following other previous applications of these techniques to event-related EEG activity^14^.

### Highlights

- TMS-EEG allows probing of human brain excitability and functionality in health and disease.
- Tensor decomposition to identify key features of high-dimensional EEG data.
- Using this data-driven approach, we reveal the effects of antiepileptic drugs on TMS-EEG.

## Supplementary information

Supplementary information

## Data Availability

Data and code are available upon request.
