# Supplementary material for: Tensor decomposition of TMS-induced EEG oscillations reveals data-driven profiles of antiepileptic drug effects
Source: Sci Rep. 2019 Nov 19;9:17057. doi: 10.1038/s41598-019-53565-9 (PMC6864053; doi:10.1038/s41598-019-53565-9)
Supplement: Supplementary file 1 — Supplementary information [file 41598_2019_53565_MOESM1_ESM.docx]

**Supplementary material**

**Tensor decomposition of TMS-induced EEG oscillations reveals data-driven profiles of antiepileptic drug effects**

Tangwiriyasakul C.^1^*, Premoli I.^1^*, Spyrou, L.^2^, Chin R.F.^3^, Escudero J^2**^, and Richardson M.P.^1**^

^1^ Department of Basic and Clinical Neuroscience, Institute of Psychiatry, Psychology and Neuroscience (IoPPN), King's College London, London, UK.

^2^ School of Engineering, Institute for Digital Communications, The University of Edinburgh, Thomas Bayes Rd, Edinburgh EH9 3FG, UK

^3^ Muir Maxwell Epilepsy Centre, Centre for Clinical Brain Sciences and MRC Centre for Reproductive Health, The University of Edinburgh, 20 Sylvan Place, Edinburgh EH9 1UW, UK

**A.1 Extended method**

*A.1.1 Experimental design*

Respective dosages for of leveiracetam (LEV, 3000 mg) and lamotrogine (LTG, 300 mg) were chosen as the most frequently prescribed dose of each medication in patients with epilepsy ^1^. Lamotrigine blocks voltage-gated sodium channels, while levetiracetam acts by binding to synaptic vesicle membrane molecule SV2A ^2^ ^3^. Baseline pre-drug TMS-EEG recordings were obtained. Subsequently, participants orally ingested a single dose of either lamotrigine, levetiracetam, or a placebo. Post-drug recordings were performed two hours after drug ingestion and blood samples for plasma drug levels were taken five minutes prior to TMS-EEG testing. Two hours was chosen as an appropriate time period for each drug to reach peak effect after intake, based on their known pharmacokinetics ^1^. Each subject participated in three experimental sessions in total, administered lamotrigine, levetiracetam or placebo in each session in a randomized order, spaced at least one week apart to allow a washout period.

*A.1.2 TMS-EMG recording*

A figure of eight coil (wing diameter 90mm) connected to a Magnetic stimulator (Magstim 200^2^) with a monophasic current wave-form was used to stimulate the left Motor Cortex (M1). An ideal coil position to produce motor evoked potentials (MEPs) in the first dorsal interosseus (FDI) muscle of the right hand was determined, continuously generating stable responses at amplitude of $\sim$1 mV TMS. This “hotspot” position and the edge of the coil wing was clearly marked using a pen on the EEG cap.

MEP recordings were obtained through surface EMG, via Ag-AgCl cup-electrodes in a belly-tendon montage. The position of the coil, with the handle pointing backwards over the scalp, away from the midsagittal line, induced a current flow in the lateral-posterior to medial-anterior route which transsynaptically activated the corticospinal system, optimal for eliciting MEPs ^4^. Resting motor threshold (RMT) was determined via the application of single TMS pulses, adopting the relative frequency method ^5^ in a fully relaxed FDI muscle, as the lowest stimulus intensity to generate an MEP of >50$\mu$V in a peak-to-peak amplitude manner in at least 5 out of 10 trials.

*A.1.3 Experimental protocol*

EEG (BrainAmp MR Plus amplifiers, Brain Products) was utilized to record brain oscillations induced by TMS. Continuous brain activity was recorded by 61 electrodes situated on an elastic cap (EasyCap 64Ch, Brain Products), and the EEG signal was digitized at a sampling frequency of 5 kHz. For all electrodes, impedance was kept at <10kΩ for the entirety of the experiment. The reference electrode corresponds to FCz and is mounted on the Brain Products EEG cap

During TMS-EEG recordings, subjects were seated in a comfortable chair, and asked to stay awake with eyes open. During TMS-EEG sesssions before and after drug intake, 150 TMS pulses were administered to the FDI hotspot over the left primary motor cortex at 100% RMT intensity. In the post-drug sessions, when a change in RMT occurred, two blocks of TMS-EEG measurements at the adjusted and un-adjusted stimulation intensities were recorded. With the aim to propose the implementation of tensor decomposition on TMS-EEG data in a simple and easy-to-apply framework, we here report non-adjusted data. Random interval variation between single TMS pulses was approximately 20%, about 4s between each trial, to reduce anticipation of TMS pulse. Throughout the TMS-EEG experiment, a masking noise was applied via headphones to reduce auditory potentials evoked by TMS coil “click” sound, which would interfere with EEG recordings ^6^ .

**A.2 Core consistency diagnostic (CORCONDIA)**

The core consistency diagnostic (CORCONDIA) is a heuristic proposed by Bro and Kiers (2003) to help gauge what number of components (*n*) may be appropriate for a given PARAFAC decomposition.

CORCONDIA assesses the appropriateness of the purely multilinear PARAFAC model to represent the data. Let’s recall that PARAFAC assumes the following model:

$$w_{ijk}\approx\sum_{r=1}^{n} a_{ir}\cdot b_{jr}\cdot c_{kr}. (A.1)$$

This can also be seen as:

$$w_{ijk}\approx\sum_{p=1}^{n} \sum_{q=1}^{n} {\sum_{r=1}^{n} g_{pqr}\cdot a}_{ip}\cdot b_{jq}\cdot c_{kr}, (A.2)$$

where

$$g_{pqr}=\left\{ \begin{matrix} 1\mathrm{if}p=q=r, \\ 0\mathrm{otherwise}. \end{matrix} \right. (A.3)$$

That is, the core tensor with elements *g_pqr_* forces the interactions to be only among componentes with the same index *p*=*q*=*r*.

The key idea behind CORCONDIA is that, once the PARAFAC model has been computed, the component matrices **A**, **B** and **C** will used in a Tucker3 as follows:

$$w_{ijk}\approx\sum_{p=1}^{n} \sum_{q=1}^{n} {\sum_{r=1}^{n} t_{pqr}\cdot a}_{ip}\cdot b_{jq}\cdot c_{kr}, (A.4)$$

where the Tucker3 core tensor *t_pqr_* is computed as the regression of the original data (**W**) onto the subspaces defined by the PARAFAC component matrices **A**, **B** and **C**. The Tucker3 core tensor with elements *t_pqr_* would contain the perfect fit of the data onto those compoentes and it can have non-zero values at any position. In contrast, the PARAFAC model constraints its core tensor *g_pqr_* to be supradiagonal ^7^.

Hence, the similarity between those two core tensors, the estimated *t_pqr_* and the ideal *g_pqr_*, can be used to measure the degree of superdiagonality of the hypothesised core tensor in PARAFAC. If the model is perfectly multilinear, the hypotethical core tensor in PARAFAC will be supradiagonal, and *t_pqr_* and *g_pqr_* will be identical. This will result in a CORCONDIA value of 100%. As *t_pqr_* and *g_pqr_* start to differ, the CORCONDIA value starts to decrease and it could eventually become negative. For a PARAFAC model with *n* components, the CORCONDIA value is computed:

$$CORCONDIA=100\left( 1-\frac{\sum_{p=1}^{n} \sum_{q=1}^{n} \sum_{r=1}^{n} \left( g_{pqr}-t_{pqr} \right)^{2}}{F} \right), (A.5)$$

which compares the distrivution of values in *t_pqr_* and *g_pqr_*, with *F* being the sum of the squares of the elements *t_pqr_* ^7^.


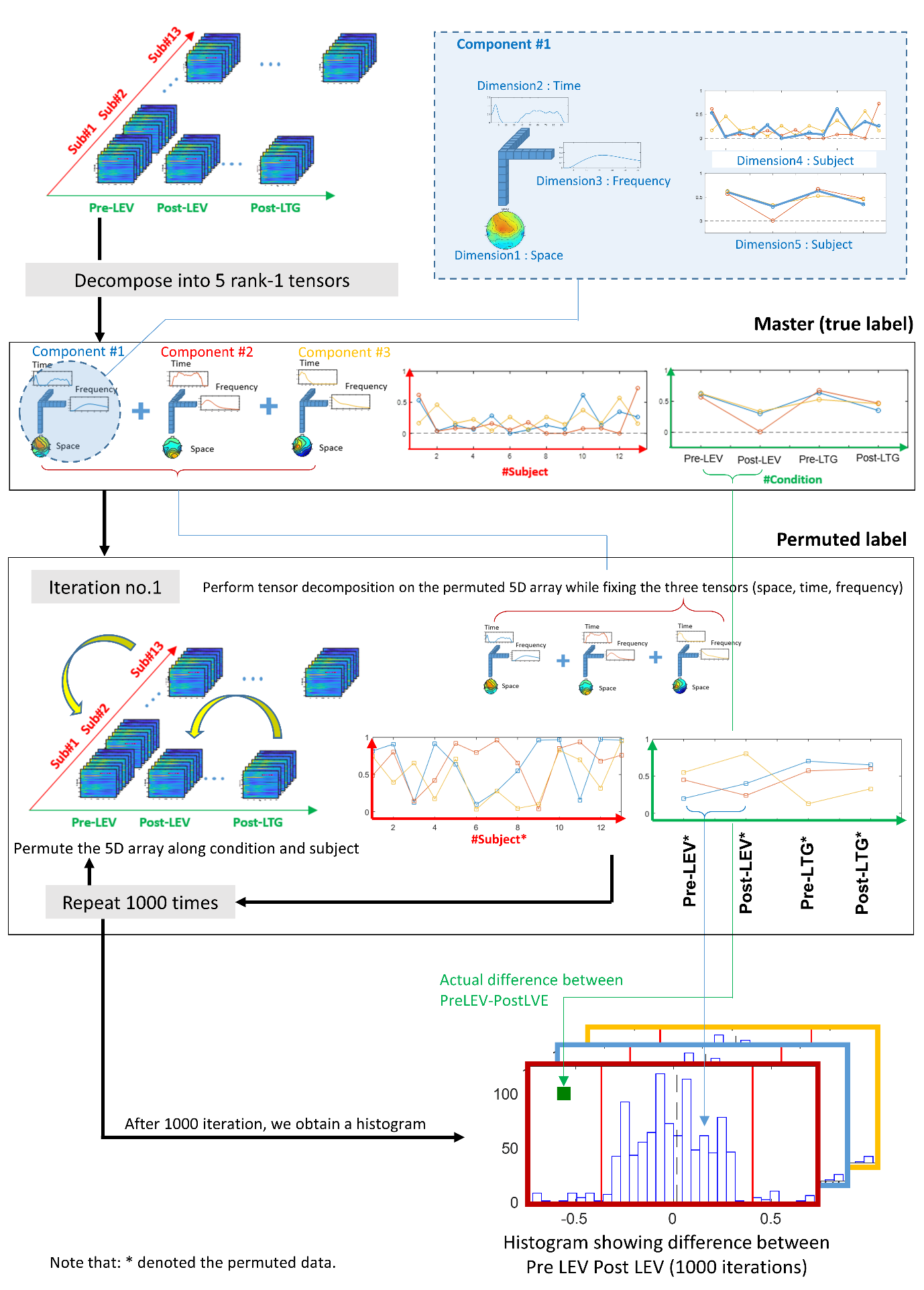


*Figure A1: Pipeline of statistical analysis*


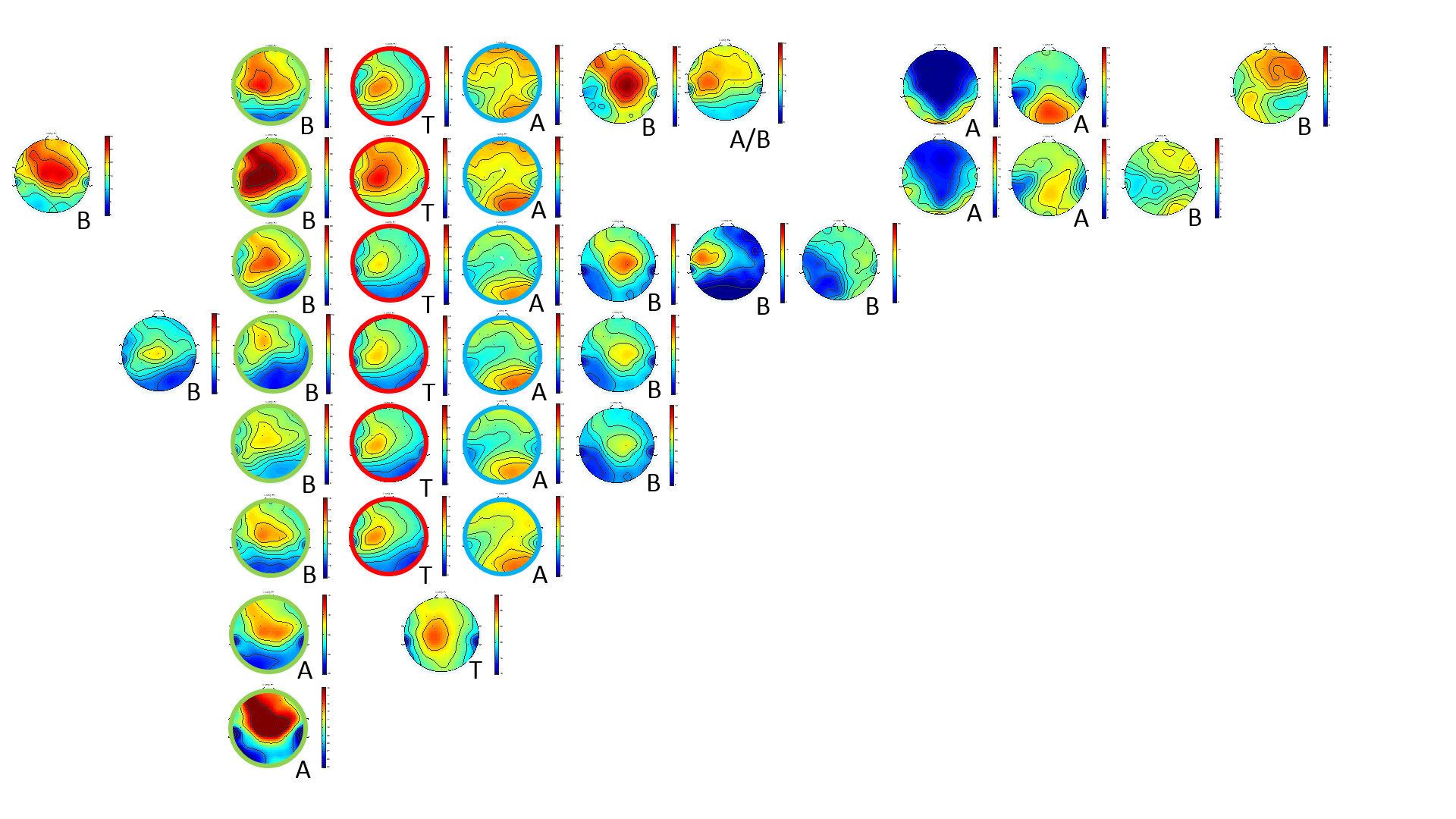


*Figure A2: Each row represents topoplots of decomposed components from eight different scenarios when the number of decomposed components was varied from one to eight (bottom to top row). Here, we found three inherited spatial patterns (highlighted in green, red and blue). These patterns are found in most scenarios (at least 6 out of 8). Note that A, B, and T denote the corresponding band (A=alpha, B=Beta, and T=Theta) in the frequency domain (2^nd^ dimension).*


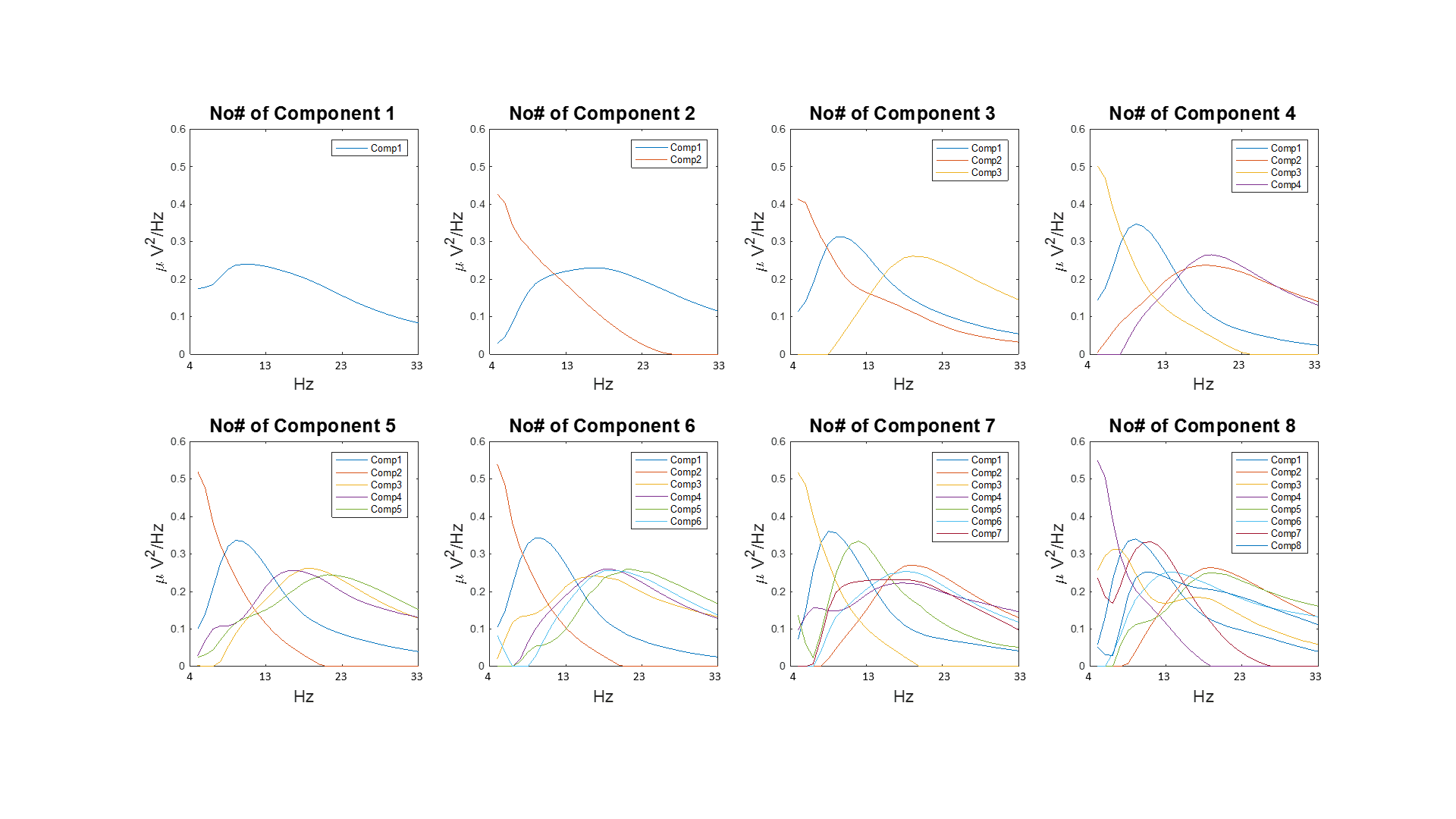


*Figure A3: Each subplot shows power spectra (2^nd^ dimension) of decomposed component(s) from eight different scenarios when a number of decomposed components was varied from one to eight.*


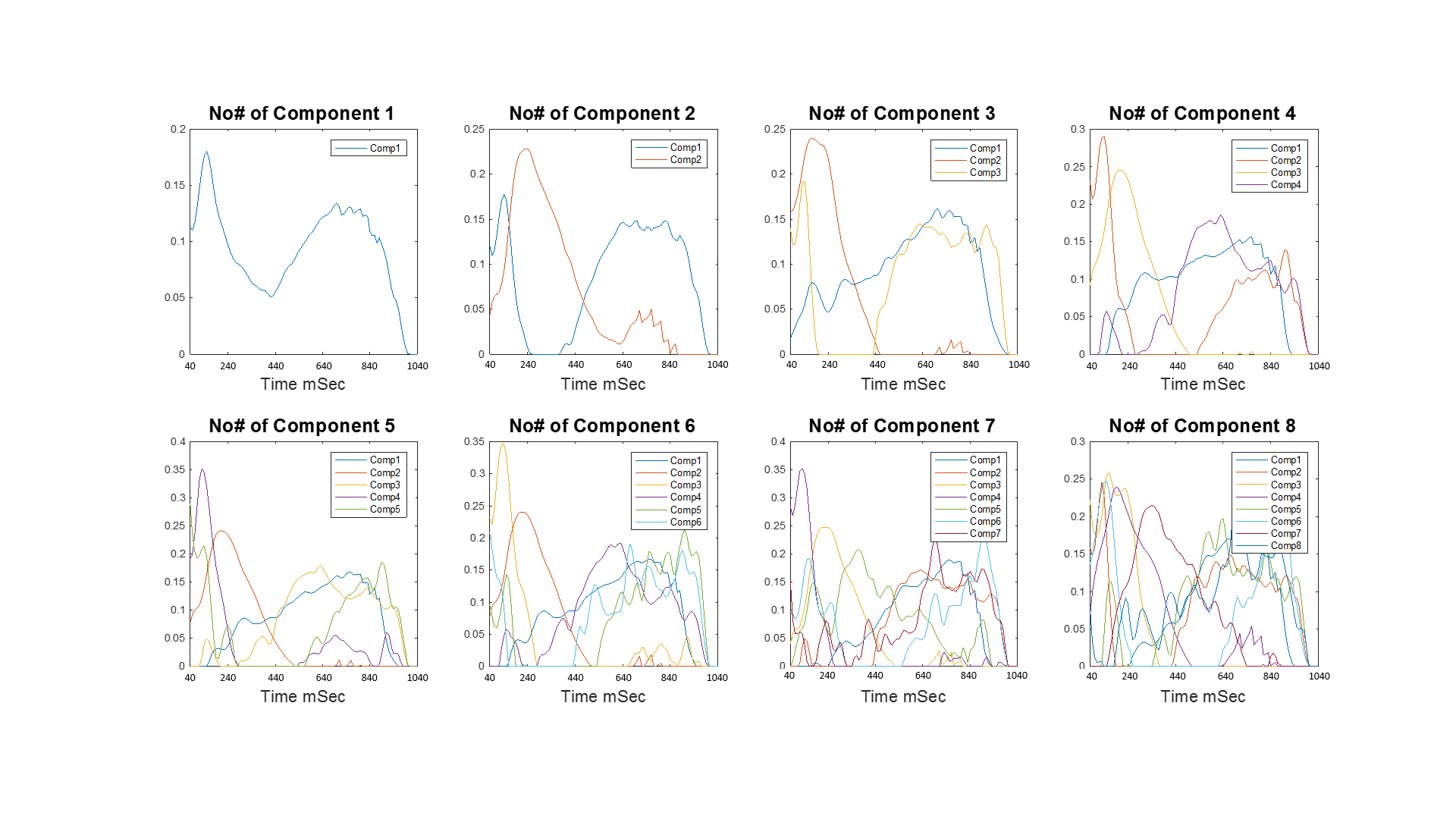


*Figure A4: Each subplot shows temporal strength (3^rd^ dimension) of decomposed components from eight different scenarios when a number of decomposed components was varied from one to eight.*

Table A1: P-values from the permutation test

|  | **Theta** | | **Alpha** | | **Beta** | |
| --- | --- | --- | --- | --- | --- | --- |
| **Model2** | Post LEV | Post LTG | Post LEV | Post LTG | Post LEV | Post LTG |
|  | 0.236 | 0.428 | **0.01*** | 0.194 | 0.217 | 0.262 |
| **Model3** | Post Placebo | Post LEV | Post Placebo | Post LEV | Post Placebo | Post LEV |
|  | 0.308 | 0.178 | 0.364 | **0.015*** | 0.363 | 0.195 |
| **Model4** | Post Placebo | Post LTG | Post Placebo | Post LTG | Post Placebo | Post LTG |
|  | 0.400 | 0.129 | 0.433 | **0.021*** | 0.086 | 0.534 |

Note that: * Significant (P<0.025)

Table A2: Percentage of CORCONDIA by a number of decomposed components.

| **Scenario** | **No. of** | **CORCODIA** |
| --- | --- | --- |
|  | **Components** |  |
| I | 1 | 100.00 |
| II | 2 | 1.14 |
| III | 3 | 0.42 |
| IV | 4 | -0.03 |
| V | 5 | 0.01 |
| VI | 6 | 0.00 |
| VII | 7 | 0.00 |
| VIII | 8 | 0.00 |

References

1 Heidegger, T., Krakow, K. & Ziemann, U. Effects of antiepileptic drugs on associative LTP-like plasticity in human motor cortex. *The European journal of neuroscience* **32**, 1215-1222, doi:10.1111/j.1460-9568.2010.07375.x (2010).

2 Cheung, H., Kamp, D. & Harris, E. An in vitro investigation of the action of lamotrigine on neuronal voltage-activated sodium channels. *Epilepsy Res* **13**, 107-112 (1992).

3 Lynch, B. A. *et al.* The synaptic vesicle protein SV2A is the binding site for the antiepileptic drug levetiracetam. *Proc Natl Acad Sci U S A* **101**, 9861-9866, doi:10.1073/pnas.0308208101 (2004).

4 Di Lazzaro, V., Ziemann, U. & Lemon, R. N. State of the art: Physiology of transcranial motor cortex stimulation. *Brain Stimul* **1**, 345-362, doi:10.1016/j.brs.2008.07.004 (2008).

5 Groppa, S. *et al.* A practical guide to diagnostic transcranial magnetic stimulation: report of an IFCN committee. *Clin Neurophysiol* **123**, 858-882, doi:10.1016/j.clinph.2012.01.010 (2012).

6 Massimini, M. *et al.* Breakdown of cortical effective connectivity during sleep. *Science* **309**, 2228-2232, doi:10.1126/science.1117256 (2005).

7 Bro, R. & Kiers, H. A. L. A new efficient method for determining the number of components in PARAFAC models. *Journal of Chemometrics* **17**, 274-286, doi:10.1002/cem.801 (2003).
